# Serum plasminogen activator inhibitor-1 levels in patients with major depressive disorder vs. healthy controls: a systematic review and meta-analysis

**DOI:** 10.47626/2237-6089-2021-0338

**Published:** 2023-08-04

**Authors:** Mohamed Elsayed, Khaled A. Mohamed, Khaled T. Dardeer, Dalia K. Zaafar, Soha Osama Hassanin, Ramy Abdelnaby, Carlos Schönfeldt-Lecuona

**Affiliations:** 1 Department of Psychiatry and Psychotherapy III University of Ulm Ulm Germany Department of Psychiatry and Psychotherapy III, University of Ulm, Ulm, Germany.; 2 Faculty of Medicine Cairo University Cairo Egypt Faculty of Medicine, Cairo University, Cairo, Egypt.; 3 Faculty of Pharmacy Modern University for Technology and Information Cairo Egypt Faculty of Pharmacy, Modern University for Technology and Information (MTI), Cairo, Egypt.; 4 Department of Biochemistry Faculty of Pharmacy MTI Cairo Egypt Department of Biochemistry, Faculty of Pharmacy, MTI, Cairo, Egypt.; 5 Department of Neurology RWTH Aachen University Aachen Germany Department of Neurology, RWTH Aachen University, Aachen, Germany.

**Keywords:** Plasminogen activator inhibitor-1, SERPINE1, major depression, meta-analysis, tissue plasminogen activator, BDNF

## Abstract

**Introduction:**

Major depressive disorder (MDD) is a severe mental health condition that affects millions of people worldwide. Etiologically, several factors may play a role in its development. Previous studies have reported elevated plasminogen activator inhibitor-1 (PAI-1) levels in patients with depression, suggesting that PAI-1 levels might be linked to the etiology of MDD.

**Methods:**

We systematically searched the following online databases: MEDLINE, Scopus, and Web of Science up to September 10, 2020, to identify studies in which PAI-1 levels were reported in subjects with MDD. Subsequently we used RevMan 5.3 to perform a meta-analysis of data extracted from the included studies using Preferred Reporting Items for Systematic Reviews and Meta-Analyses (PRISMA) and PICO criteria for the search and analysis.

**Results:**

Six studies that reported mean ± standard deviation (SD) were included in the analysis, with a total of 507 MDD patients and 3,453 controls. The overall standardized mean difference (SMD) was 0.27 (95% confidence interval [95% CI] 0.01-0.53). PAI-1 serum levels were 0.27 SDs higher in MDD patients than in controls. The test for overall effect was significant (z = 2.04, p = 0.04). Substantial heterogeneity was detected among the studies, demonstrated by the inconsistency test (I^2^ = 72%) and the chi-square test (χ^2^ = 18.32; p = 0.003).

**Conclusion:**

This systematic review and meta-analysis showed that MDD might be related to elevated PAI-1 levels. We propose larger prospective clinical studies to further investigate this clinical correlation and validate the clinical significance of these observations.

## Introduction

Major depressive disorder (MDD) is a primary cause of morbidity and significantly affects the productivity of society.^1^ The lifetime prevalence of MDD is approximately 10%,^2^ imposing an economic burden of almost 92 billion Euros annually in Europe.^2^ MDD impacts personal and family life negatively and is considered a risk factor for severe disorders, for example, Alzheimer’s dementia,^3^ cardiovascular illness,^4^ and dependence on alcohol and other drugs. MDD affects emotional functioning and quality of life,^5^ and therapy-resistant MDD is associated with high suicide incidence.^6^ The etiology is heterogeneous and clinical presentation, course of illness, and treatment response can differ considerably. Its pathophysiology is linked to altered neurocircuit activity, deficient monoamines, neurotrophic alterations, glucocorticoid dysregulation, immuno-inflammation, disturbed energy metabolism, imbalance in the microbiome, and oxidative stress.^7 - 10^ The exact underlying etiological pathomechanisms remain unknown and treatments are not always effective. Previous studies have reported that serum plasminogen activator inhibitor-1(PAI-1) levels were increased in patients with MDD and anxiety, suggesting that pAI-1 could play a vital role in MDD pathophysiology and stress response.^11 - 14^

Tissue plasminogen activator (tPA) is a major plasminogen activator that converts inactive plasminogen into active plasmin. Active plasmin breaks down fibrin clots, helping to restore vascular patency.^15^ PAI-1 is a critical inhibitor of tPA,^14^ and it was reported that low tPA levels are related to increased levels of its inhibitor PAI-1.^14^ The mechanism by which low tPA levels are related to MDD^16^ might involve brain-derived neurotrophic factor (BDNF). The precurser of brain-derived neurotrophic factor (proBDNF) is proteolytically cleaved into mature BDNF (mBDNF) by tPA and by plasmin (the end product of tPA)^16^ and promotes neuronal apoptosis and long-term depression, while mBDNF has anti-apoptotic properties and favors long-term potentiation.^17^ Moreover, physical exercise has antidepressant effects and increases tPA and mBDNF levels.^12^ The inability to convert proBDNF into mBDNF is associated with depression pathogenesis and, consequently, mBDNF is implicated in the mechanism of action of antidepressants.^18^

**Figure 1:**
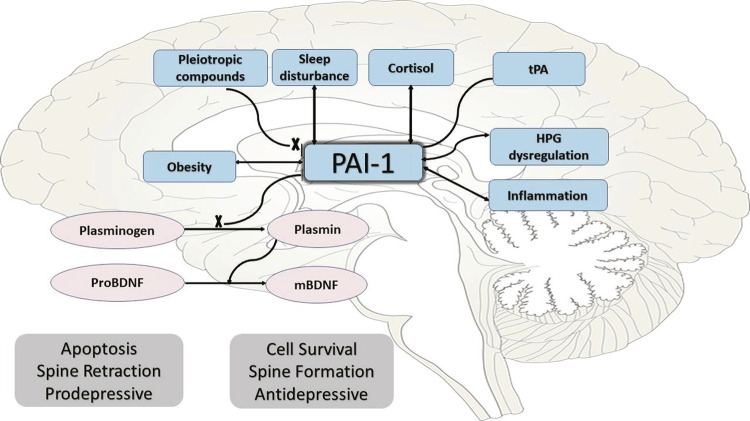
Plasminogen activator inhibitor-1 (PAI-1) and its association with major depressive disorder (MDD). HPG = hypothalamus-pituitary-gonadal; mBDMF = mature brain-derived neurotrophic factor; proBDNF = pro-BDNF; tPA = tissue plasminogen activator.

Figure 1 shows the association between PAI-1 and MDD as different factors affecting PAI-1, including pleiotropic compounds, sleep disturbances, cortisol dysregulation, tPA level, hypothalamus-pituitary-gonadal (HPG)-axis dysregulation, and inflammation. The PAI-1 inhibits activation of plasminogen into plasmin which in turn leads to inhibition of conversion proBDNF into its mature form, mBDNF. Therefore, PAI-1 enhances apoptosis, spine retraction, and depression.

**Figure 2:**
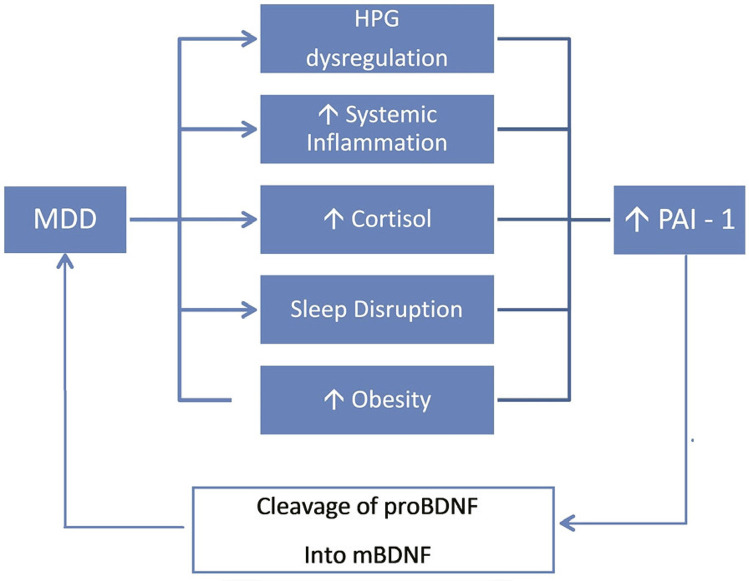
Plasminogen activator inhibitor-1 (PAI-1) and its association with mature brain-derived neurotrophic factor (mBDNF). HPG = hypothalamus-pituitary-gonadal; MDD = major depressive disorder; proBDNF = pro-BDNF.

Figure 2 shows the association between PAI-1 and mBDNF. HPG-axis dysregulation, increased systemic inflammation, cortisol level, sleep disurbance, and obesity all increase PAI-1 levels. Elevated PAI-1 levels can cause MDD by inhibiting conversion of pro-BDNF into mBDNF.

Considering the reported data on the effects of tPAI on MDD pathophysiology and treatment resistance ( Figure 1 ) and the lack of strong evidence on this topic, the authors decided to conduct a systematic review of existing studies in this field to provide a holistic, comprehensive conclusion on the impact of PAI-1 levels on depression. This study aims to examine and systematically review and analyze the literature and the reported results related to the impacts of PAI-1 levels on the pathophysiology of MDD and treatment options.

## Methods and materials

### Data sources and searches

We systematically searched the MEDLINE (through PubMed), Scopus, and Web of Science online databases up to September 10, 2020, in order to identify studies that assess PAI-1 levels in MDD subjects using the following MeSH terms: (“SERPINE1” OR “Serpin E1” OR “PAI-1” OR “Type 1 Plasminogen Activator Inhibitor” OR “Plasminogen Activator Inhibitor-1”) AND (“depression” OR “depressive”). We included the studies in accordance with the Preferred Reporting Items for Systematic Reviews and Meta-Analyses (PRISMA). We applied no study design or year of publication search filters. We exported the search results to EndNote X9.1 (Clarivate Analytics, https://clarivate.com/) to remove duplicate studies. Next, the studies were exported to an Excel spreadsheet and screened by title and abstract by three independent reviewers (KA, RA, MS). Any disagreements about inclusion or exclusion of the screened studies were resolved by a senior study member.

### Data extraction

**Figure 3:**
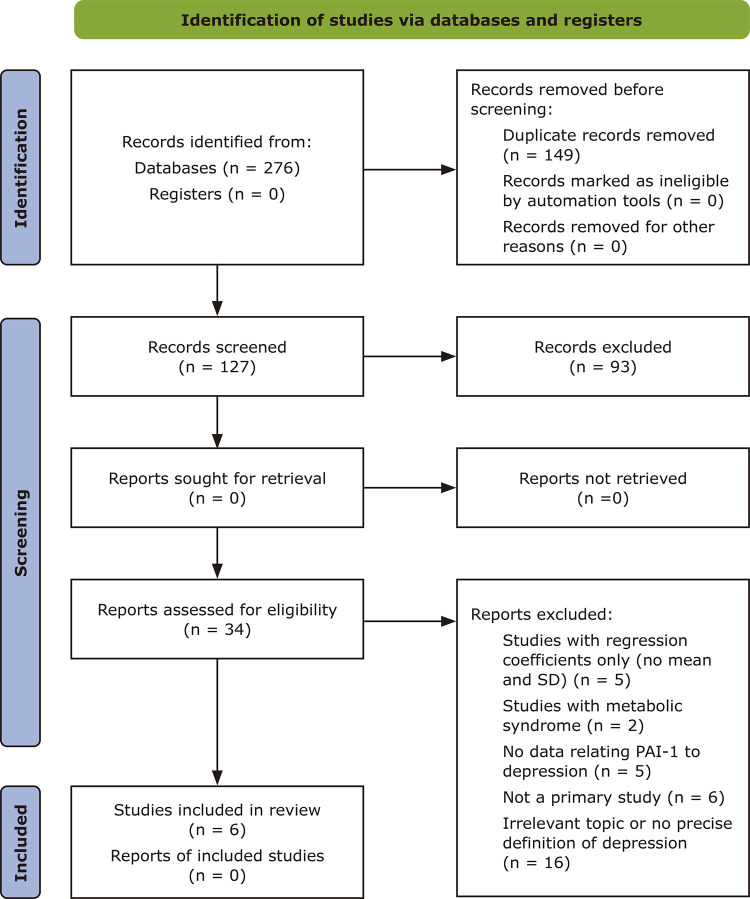
Preferred Reporting Items for Systematic Reviews and Meta-Analyses (PRISMA) flow diagram illustrating selection of studies to be included in the systematic review and meta-analysis. PAI-1 = plasminogen activator inhibitor-1; SD = standard deviation.

Data extraction was conducted by two independent authors. We extracted the summary data and statistical data to a Microsoft Excel spreadsheet template. More than one author checked the data extracted. The details of the searches and selection and inclusion of the studies are summarized in the following PRISMA diagram ( Figure 3 ).

### Study selection

Studies reporting PAI-1 levels in depressed human subjects published in English or German in international peer-reviewed journals were included. Studies reporting mean ± standard deviation (SD) were included in the quantitative analysis. We excluded studies that reported other variables such as correlation or regression coefficients or that did not report a clear relationship between MDD and PAI-1, or that did not include a precise definition of depression. We also excluded studies of psychiatric disorders not related to MDD, studies assessing the relation between genetics and depressive disorders, or animal studies reporting PAI-1.

### Quality assessment of the included studies

The methodological quality of the included studies was assessed independently and discussed by two authors (ME, KTD) using the Newcastle-Ottawa-Scale (NOS). The NOS contains three main categories: patient selection, group comparability, and outcome assessment. It has nine questions allowing a maximum score of 9. Studies were considered to be of high (scored 7-9), moderate (scored 4-6), or low quality (scored 0-3). In addition, a modified version of the National Institutes of Health-Study Quality Assessment Tool for Observational Cohort and Cross-Sectional Studies (NIH-SQAT) was also used. This tool consists of 14 questions and allows three overall ratings according to the total score; “good”, “fair”, or “poor”.

### Data analysis

RevMan 5.3 was used in the meta-analysis of data.^19^ Five studies^20 - 24^ reported mean ± SD, and one study^25^ reported mean and range, so these data were transformed to mean and SD values using the method described by McGrath et al.^19^ Eskandari et al.^20^ reported two measurements of PAI-1 performed at 8:00 am and 8:00 pm; the average of both was calculated. Malan et al.^21^ reported the measurements of men and women separately; so the average of both measurements was taken, to be consistent with the other studies. SD was obtained from a 95% confidence interval (95%CI) using the method described in the Cochrane Handbook for Systematic Reviews of Interventions.^22^ Standardized mean difference (SMD) was used as an overall effect measure. The studies were weighted by inverse variance, and a random-effects model was chosen owing to the heterogeneity of the data. Inconsistency (I^2^), chi-square (χ^2^), and tau-square tests were performed to test for heterogeneity. A p-value less than 0.05 was considered statistically significant; I^2^ > 50% was considered indicative of substantial heterogeneity in the studies.^22^

### Sensitivity analysis

To examine the effect of a single study on the overall effect, a leave-one-out analysis was conducted. We also excluded an outlying study to examine the pooled effect and to account for heterogeneity.

### Publication bias analysis

Egger’s test and a funnel plot were employed to assess publication bias in the included studies.

## Results

### Study characteristics

Following screening, a total of 34 studies were assessed for eligibility and six studies were chosen for inclusion in the meta-analysis. Detailed methodology and reasons for exclusion are presented in the PRISMA flow diagram ( Figure 3 ). The characteristics of included studies and clinical data for both MDD and healthy control groups are presented in Tables 1 , 2 , and 3 . The serum PAI-1 values are shown in Table 4 .

**Table 1:** Characteristics of the studies included in the meta-analysis

Author	Year	Country	Tools used to assess depression
Eskandari ^20^	2005	United States	DSM-IV criteria & HAMD scale
Lahlou-Laforet ^26^	2006	France	CES-D scale
Schroeder ^25^	2007	Switzerland	DSM-IV criteria & HADS & ADS-L scales
Pan ^23^	2008	China	CES-D
Jiang ^24^	2016	China	DSM-IV criteria & HDRS scale
Malan ^21^	2016	South Africa	PHQ-9

ADS-L = Allgemeine Depressions-Skala-L; CES-D = Center for Epidemiologic Studies Depression Scale (20-item); DSM-IV = Diagnostic and Statistical Manual of Mental Disorders, 4th Edition; HADS = Hospital Anxiety and Depression Scale; HAMD = Hamilton Depression Scale (HAM-D, 24 questions); HDRS = Hamilton Depression Rating Scale; PHQ-9 = Patient Health Questionnaire (9-item self-report).

**Table 2:** Characteristics of the populations in the studies included

Author	Population and study design	Exclusion criteria	PAI-1 Measurement	Main findings	Adjustment
Eskandari 2005 ^20^	A prospective study of 45 women with MDD diagnosed by DSM-IV criteria and 25 controls with no history of DSM-IV diagnosis. Severity of depression was assessed with the HAM-D scale.	Menopausal women, endocrine disorders, patients with potential suicidal risk, subjects with a current or past history of eating disorders, bipolar disorders, schizophrenia, or schizoaffective disorder were excluded.	Blood samples were collected at 8:00 pm following ad libitum diet and fasting blood samples were collected at 8:00 am of the following morning once per hour till 8:00 pm, for a total of 25 samples.	PAI-1 level was higher in patients with MDD.	Seventeen MDD patients and 17 age, race, BMI, and smoking-matched controls
Lahlou-Laforet 2006 ^26^	A comparative study with 231 men aged from 40 to 65, 123 with no CHD and 108 with CHD. Depression was assessed with the CES-D scale.	Patients with recent myocardial infarction or unstable angina during the previous 3 months or a prescription for thrombolytic agents during the same period	PAI-1 measurements were done between 8:00 and 10:00 am to control for circadian variations.	PAI-1 Activity was higher in blood samples of depressed patients	Forty-nine depressed patients and 182 non-depressed patients with adjustment for CHD status, hypertension, triglyceride concentration, smoking, BMI, tPA antigen and fibrinogen levels
Schroeder 2007 ^25^	A comparative study with 306 sequentially enrolled participants, 214 with CAD and 92 without CAD. Diagnosis of MDD was based on DSM-IV criteria. The HADS and ADS-L scales were used as screening instruments for depression and anxiety. The HAMD scale was used to assess severity of depression.	Insufficient knowledge of the German language and incomplete answering of questionnaires were the only criteria listed for exclusion.	No detailed information was available regarding method of sample collection.	PAI-1 was non-significantly higher in MD patients than participants with negative screening by HADS and ADS-L scales.	Thirty MDD patients and 161 with negative screening. The population was split into CHD patients and controls without CHD and coagulation and fibrinolytic measures were assessed between MD patients and patients with negative screening within each group.
Pan 2008 ^23^	A cross-sectional study with 3,289 community participants aged 50-70 years old; 312 depressed and 2,977 with no depression symptoms. Depressive symptoms were assessed using the CES-D scale with a cut-off point of 16.	Patients with self-care disabilities, severe psychological disorders, newly diagnosed with cancer, coronary heart disease, stroke, Alzheimer’s disease and dementia within the 6 month period before the start of the study, or currently diagnosed with tuberculosis, AIDS, or other communicable diseases	Blood samples were collected in the morning after 8 hours of fasting.	No statistical difference in PAI-1 level between depressed and non-depressed patients.	Adjusting for age, sex, BMI, smoking status, drinking status, physical activity level, educational level, geographic location, residential area, comorbidity status, log-insulin, log-triglyceride, log-total cholesterol, log-systolic blood pressure, and use of anti-inflammatory drugs.
Jiang 2016 ^24^	A comparative study with 17 MDD inpatients diagnosed according to DSM-IV criteria. Patients were drug free for 2 weeks. HDRS was used to assess severity of depression. Seventeen healthy controls with no history of DSM-IV axis I or II disorders and HDRS scores < 7.	Comorbid Axis I diagnosis, alcohol or substance abuse or dependence, or other neurological illnesses, including dementia or stroke	Blood samples were collected between 6:30-8:00 am.	Serum PAI-1 concentration was significantly higher in MDD patients than controls.	Adjusting for age, education, and body weight.
Malan 2017 ^21^	A cross sectional study with 181 black African urban-dwelling teachers aged 25-60 years. Eighty-two patients with depression symptoms and 99 controls without depression. Depressive symptoms were assessed with the PHQ-9 scale.	Pregnancy, lactation, psychotropic substance users, tympanum temperature higher than 37.5ºC, individuals vaccinated or who donated blood in the 3 months prior to participating and HIV infection	Blood samples were obtained before 9:00 am after fasting overnight.	PAI-1 levels were significantly higher in men with depression symptoms than those without. However, no statistical difference was found in women.	Adjusting for age, cotinine, BMI, GGT, physical activity, HDL-cholesterol, mean arterial pressure, and statin use. Where PHQ is 10 or greater, moderately depressed; PHQ lower than 10, no apparent depression.

ADS-L = Allgemeine Depressions-Skala-L; AIDS = acquired immune deficiency syndrome; BMI = body mass index; CAD = coronary artery disease; CES-D = Center for Epidemiologic Studies Depression Scale (20-item); CHD = coronary heart disease; DSM-IV = Diagnostic and Statistical Manual of Mental Disorders, 4th Edition; GGT = gamma-glutamyl transferase; HADS = Hospital Anxiety and Depression Scale; HAMD = Hamilton Depression Scale (HAM-D, 24 questions); HDRS = Hamilton Depression Rating Scale; HIV = human immunodeficiency virus; MDD = major depressive disorder; PAI-1 = plasminogen activator inhibitor-1; PHQ-9 = Patient Health Questionnaire (9-item self-report); tPA = tissue plasminogen activator.

**Table 3:** - Basic clinical data for the subjects included in the meta-analysis

Variable	Patients	Scale score	Disease duration	No. of episodes	Controls
Eskandari 2005 ^20^					
n (f/m)	45 (45/0)	9 ± 7.6	67 ± 73 (n = 34)	4 ± 3 (n = 34)	28 (28/0)
Age (years)	37 ± 6.8				35 ± 6.5
Weight (kg)	75.3 ± 17.2				67.9 ± 10.2
BMI (kg/m^2^)	27.5 ± 6.3				24.1 ± 3.4
Smoking	2.8 ± 5.6 (pack/year)				2.2 ± 5.4 (pack/year)
Lahlou-Laforet 2006 ^26^					
n (f/m)	49	-	-	-	182
	Other clinical data are reported in the study according to presence of CHD				Other clinical data are reported in the study according to presence of CHD
Schroeder 2007 ^25^					
n (f/m)	30 (10/20)	CES-D score > 17	-	-	173 (131/42)
Age (years)	65 (42-78)*				65 (30-88)*
Weight (kg)	-				-
BMI (kg/m^2^)	5 : 14 : 11 (< 25 : 25-30 : > 30)				50-81-32 (< 25 : 25-30 : > 30)
Smoking	3 : 17 : 10 (yes : past : no)				10 : 57 : 33 (yes : past : no)
Pan 2008 ^23^					
n (f/m)	312 (214/98)	-	-	-	2,977 (1,397/1,580)
Age (years)	58.26 ± 6.17				58.64 ± 5.99
Weight (kg)	-				-
BMI (kg/m2)	24.85 ± 3.9				24.42 ± 3.55
Smoking	77 (current)				843 (current)
Jiang 2016 ^24^					
n (f/m)	17 (14/3)	21.6 ± 6.3	44.5 ± 70.0	1.5 ± 0.9	17 (13/4)
Age (years)	48 ± 12				54 ± 3
Weight (kg)	62 ± 15				67 ± 7
BMI (kg/m^2^)	-				-
Smoking	-				-
Malan 2017 ^21^					
n (f/m)	82 (46/36)	-	-	-	99 (47/52)
	Other clinical data are reported in the study according to sex				Other clinical data are reported in the study according to sex

CES-D = Center for Epidemiologic Studies Depression Scale (20-item); CHD = coronary heart disease; f/m = female/male.

Data are presented as mean ± standard deviation.

* Median (interquartile range).

**Table 4:** Mean serum PAI-1 levels used in the meta-analysis

	MDD patients			Controls		
Study	Mean	SD	Subjects (n)	Mean	SD	Subjects (n)
Eskandari 2005 ^20^	9.0 µg/L	16.06	17	3.7 µg/L	6.1	17
Lahlou-Laforet 2006 ^26^	17.0*	11	49	13.4*	8.9	182
Schroeder et al. 2007 ^25^	30.8 ng/mL	22.6	30	27.4 ng/mL	27.68	161
Pan 2008 ^23^	9.07 ng/mL	11.9	312	10.32 ng/mL	23.8	2,977
Jiang 2016 ^24^	6.2 pg/mL	2	17	4.5 pg/mL	1.6	17
Malan 2017 ^21^	36.73 ng/mL	9.28	82	34.11 ng/mL	9.22	99

MDD = major depressive disorder; SD = standard deviation.

The mean represents the serum PAI-1 levels measured in patients and controls.

* PAI-1 activity.

### Quality assessment

Following the NOS scoring categories, two studies were of high quality and four studies were of moderate quality ( Table 5 ). According to the NIH-SQAT guidelines and after interrater consensus, two^21 , 23^ of the included studies were considered to be of good quality (scored 7) and four^20 , 24 - 26^ were of moderate quality (scored 6).

**Table 5:** Newcastle-Ottawa-Scale for quality assessment of included studies

	Selection (maximum:★★★★)				Comparability (maximum:★★)	Exposure (maximum:★★★)			Total score (out of 9)

	Is the case definition adequate? (maximum:★)	Representativeness of the cases (maximum:★)	Selection of controls (maximum:★)	Definition of controls (maximum:★)	Comparability of cases and controls on the basis of the design or analysis	Ascertainment of exposure (maximum:★)	Same method of ascertainment for cases and controls (maximum:★)	Non-response rate (maximum:★)	
Eskandari 2005 ^20^	★	-	★	★	★★	-	★	-	6
Lahlou-Laforet 2006 ^26^	★	-	-	★	★★	-	★	★	6
Schroeder 2007 ^25^	★	★	-	-	★★	★	★	-	6
Pan 2008 ^23^	★	★	-	-	★★	★	★	-	7
Jiang 2016 ^24^	★	-	★	★	★	-	★	★	6
Malan 2017 ^21^	★	-	★	★	★★	★	★	-	7

### Serum PAI-1 level

**Figure 4:**
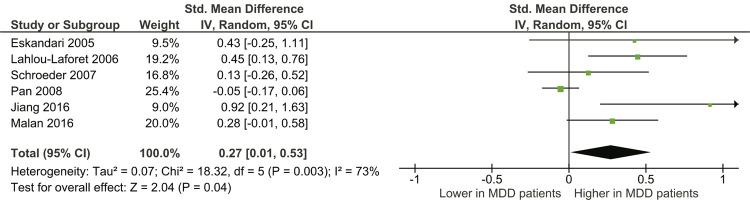
- Forest plot of plasminogen activator inhibitor-1 (PAI-1) levels in major depressive disorder (MDD) patients compared to control groups; the right side represents higher serum PAI-1 in MDD patients, or lower levels in controls, the left side represents lower levels in MDD patients, or higher levels in controls. 95%CI = 95% confidence interval; df = degrees of freedom; I2 = inconsistency test; IV = inverse variance.

Six studies^20 , 21 , 23 - 26^ that reported mean ± SD were included in the meta-analysis with a total of 507 MDD patients and 3,453 adjusted healthy controls, the overall SMD was 0.27 (95%CI 0.01-0.53); serum PAI-1 levels were 0.27 SDs higher in depression patients than controls. The test for overall effect was significant (z = 2.04, p = 0.04). Substantial heterogeneity was detected in the studies, since the I^2^ statistic was 72%, χ^2^ was 18.32 (p = 0.003) ( Figure 4 ).

### Sensitivity analysis

**Figure 5:**
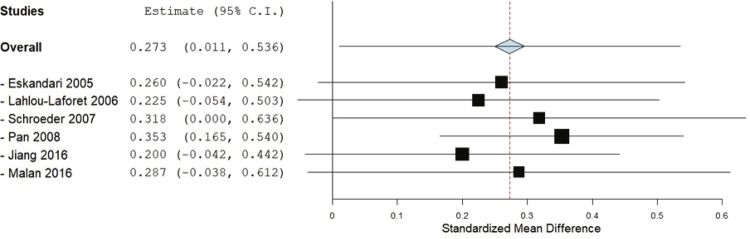
Forest plot of the leave-one-out analysis. 95%CI = 95% confidence interval.

A leave-one-out analysis revealed that no single study affected the results significantly, as the SMD was still within the CI of the overall results following removal of each study. Eliminating some studies shifted the value to non-significant, where the CI intersects with 0, as shown in Figure 5 .

We tried incorporating the 8:00 am measurements taken by Eskandari et al.,^20^ to be consistent with other studies instead of averaging the 8:00 am and 8:00 pm measurements, but doing so did not remarkably affect the result (SMD 0.28, 95%CI 0.02-0.55, p = 0.04). Eliminating Pan et al.^23^ reduces the heterogeneity level from 74 to 11% and increases the overall effect (SMD 0.36, 95%CI 0.17-0.55, p = 0.0002). However, eliminating studies on the basis of heterogeneity is not recommended, since it may introduce bias (Cochrane Handbook Chapter 10, Section 10.10.3).^22^

### Publication bias

**Figure 6:**
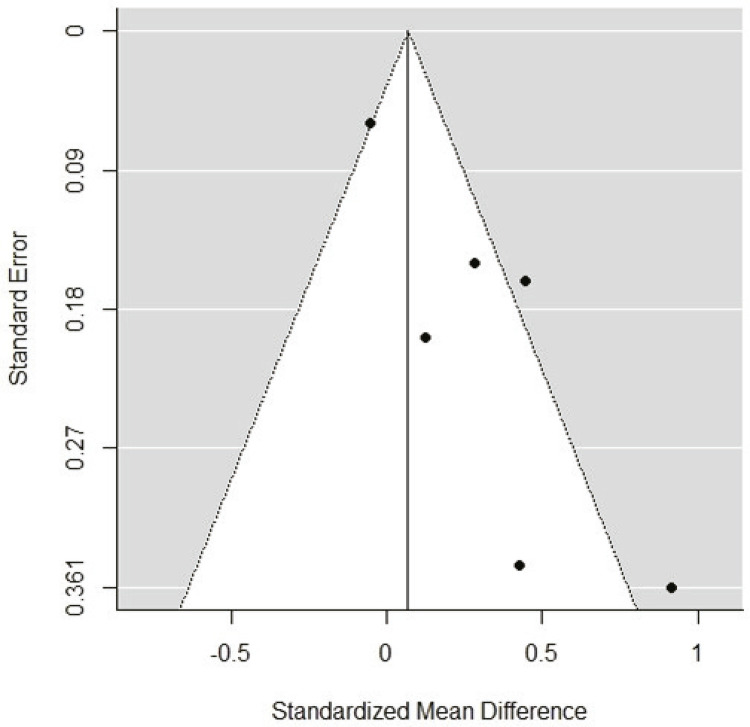
Funnel plot test for publication bias using the effect measure and the standard error.

Egger’s test for funnel plot asymmetry ( Figure 6 ) suggests presence of publication bias (p = 0.0002). However, the results of Egger’s test should be interpreted with care when a meta-analysis includes a small number of studies.^22^

## Discussion

Previous research has provided evidence that patients with MDD often have elevated serum PAI-1 levels.^11 - 14^ PAI-1 is known for its inhibitory action on tPA.^15 , 16^ Inhibition of tPA predisposes to depressive symptoms due to involvement of BDNF.^15 , 16^ Mature BDNF is proteolytically cleaved from proBNDF by tPA and plasmin, which is itself the end product of tPA.^17^ Whereas proBDNF stimulates neuronal apoptosis and depression, mBDNF has anti-apoptotic properties.^17^ Physical exercise that has antidepressant effects causes elevation of tPA and mBDNF levels.^12^ Failure of the conversion of proBDNF into mBDNF is associated with the pathogenesis of MDD. Mature BDNF is even implicated in the mechanism of action of selective serotonin reuptake inhibitor antidepressants, such as escitalopram.^18^ Therefore, this study aimed to systematically examine and analyze the results reported in the literature about the impact of PAI-1 levels on depression. To the best of our knowledge, our study is the first systematic review and meta-analysis to assess the association between PAI-1 and MDD.

In this meta-analysis, six studies^20 , 21 , 23 - 26^ were included, with a total of 3,960 participants (507 MDD patients and 3,453 controls). According to the results presented in the six studies included in the quantitative synthesis, serum PAI-1 levels were 0.27 SDs higher in MDD patients than in controls.

The six studies included in this meta-analysis focused on rating scales rather than a clinical diagnosis for defining depression. Furthermore, the six studies included did not all focus on the same subtype of depression, so that the criteria used for diagnosis were not homogenous. A large proportion of the patients with depression included in the studies suffered from cardiovascular diseases, such as coronary heart disease (CHD) in the study by Lahlou-Laforet et al.^26^ and coronary artery disease (CAD) in the study by Schroeder et al.^25^ Furthermore, the included studies focused on specific age groups. Besides, differences in episodes, duration of illness, and illness severity among patients with MDD might be a potential confounding factor. Furthermore, PAI-1 levels are modulated by angiotensin, glucocorticoids, and aldosterone. However, the levels of these factors were not consistently screened in the six studies included. Jiang et al. highlighted that in their rodent depression data model, stress increases PAI-1 expression in the medial prefrontal cortex, and the hippocampus. Also, chronic escitalopram treatment downregulated PAI-1 expression in these brain subregions and decreased the active PAI-1 concentration in the serum, but not in the CSF in rodents,^9^ while it did not affect the PAI-1 concentrations in the serum of MDD patients.^25 , 26^

Many factors are known to affect PAI-1 expression, including obesity,^2^ sleep dysregulation,^27^ and hypothalamus-pituitary-adrenal (HPA) axis dysregulation^4^ ( Figures 1 and 2 ). Also, female hormonal dysregulation affects PAI-1, which leads to an increase in the incidence of female depression.^23 , 28^ Some pleiotropic compounds also affect PAI-1 activity, including metformin,^29^ resveratrol, and other antioxidants such as curcumin, ginko biloba, shikonin, and theaflavin by many different mechanisms.^13 , 30^

Pharmacological treatments available nowadays are useful, but unfortunately, around 30-40% of patients are resistant to such treatments.^31 , 32^ Besides, antidepressants could cause side effects.^29^ Party et al. introduced a new model for depression using PAI knockout mice, which showed resistance to antidepressant therapy.^28^

Depression is known to be associated with alteration of PAI-1 protein, blood coagulation, and fibrinolysis.^33^ Tsai et al. concluded that since PAI-1 dysregulation might be a biomarker for MDD, drugs that could decrease PAI-1 levels might help treat depression.^27^ Furthermore, PAI-1 may be a mediator in which MDD could precipitate CVD development via BDNF metabolism, adiposity, sleep dysregulation, and alteration of the hypothalamic-pituitary-adrenal (HPA)-axis.^34^

Further studies on the relation between PAI and MDD should include larger samples of patients with MDD. Besides, it is warranted to standardize measurement of PAI-1 and other variables. More studies should focus on measuring PAI-1 levels in cerebrospinal fluid **(** CSF). Measurements of PAI-1 in serum and CSF would probably provide more reliable results. Intake of antidepressants might interfere with the results, as antidepressant treatment might influence PAI-1 levels.^35^ For example, in the study by Jiang et al.,^24^ escitalopram treatment significantly decreased PAI-1 levels in serum, but not in CSF. It is currently unclear whether PAI-1 can cross the blood-brain barrier,^34^ despite its presence in certain areas of the brain and the CSF.^33^

Despite the intensive search of the current literature and careful data extraction, this review still has limitations. Some studies focused only on one gender: Eskandari et al. examined only females,^20^ while Lahlou-Laforet examined only males.^26^ Eskandari et al. even focused only on premenopausal females.^20^ Several confounding factors were present in the Eskandari et al. study; patients continued their antidepressant medication, which might be a confounding factor. Lahlou-Laforet et al.’s study included confounding factors such as smoking, hypertension, triglyceride concentration, and body mass index.^26^ It is worth mentioning that each study focused on certain coagulation factors including PAI-1; the coagulation factors that were studied varied between the six studies. Furthermore, Malan et al. focused only on members of the black African race^21^ and Pan et al. focused solely on the Chinese population,^23^ while the other studies probably involved various races. Also, Malan et al. measured fasting PAI-1 levels,^21^ while the other studies did not mention whether measurements were in a fasting state or not. Furthermore, data heterogeneity and publication bias were detected, and the limited number of studies still poses a main concern.

## Conclusion

In conclusion, this systematic review and meta-analysis revealed that elevated PAI-1 is associated with MDD, however heterogeneity, publication bias, and the limited number of studies should be taken into consideration when interpreting the result. More extensive prospective clinical studies are required to thoroughly examine its clinical correlation and validate the clinical significance of these observations.
